# Endometriosis in Patients with Mayer-Rokitansky-Küster-Hauser-Syndrome—Histological Evaluation of Uterus Remnants and Peritoneal Lesions and Comparison to Samples from Endometriosis Patients without Mullerian Anomaly

**DOI:** 10.3390/jcm11216458

**Published:** 2022-10-31

**Authors:** Sahra Steinmacher, Hans Bösmüller, Massimo Granai, André Koch, Sara Yvonne Brucker, Kristin Katharina Rall

**Affiliations:** 1 Department of Women’s Health, Tuebingen University Hospital, Calwerstr 7, 72076 Tuebingen, Germany; 2 Department of Pathology, Tuebingen University Hospital, Liebermeisterstraße 8, 72076 Tuebingen, Germany

**Keywords:** Mayer-Rokitansky-Küster-Hauser syndrome, endometriosis, uterus remnants, uterus aplasia

## Abstract

Congenital Mayer-Rokitansky-Küster-Hauser (MRKH) syndrome is a Mullerian-duct anomaly that is characterized by agenesis of the uterus and upper part of the vagina. Uterus remnants of varying sizes can often be found. Although a functional uterus is missing, the existence of endometriosis in this patient group has been described in the literature; however, a histopathological comparison of the characteristics of the endometrium within the uterus remnants versus endometriotic peritoneal lesions in the same patient is lacking. Moreover, the characteristics of endometriotic tissue in patients with MRKH syndrome have not been correlated with those of patients with endometriosis without Mullerian anomaly. Patients who underwent laparoscopic neovagina creation with the removal of uterus remnants and possible resection of endometriotic lesions between 2010 and 2022 at the Department of Women’s health of the University of Tuebingen were included in our study. Uterine remnants and endometriotic tissue were evaluated via histopathology and immunohistochemistry and were compared to endometriotic samples from patients without Mullerian anomaly. Endometriosis was detected in nine MRKH patients; in four patients, endometrial remnants could be sufficiently compared to endometriotic lesions. All samples exhibited increased expression of hormonal receptors. In two patients, Ki67 proliferation index was significantly increased in peritoneal endometriotic lesions compared with the endometrium of the remnants. In contrast, endometrium and endometriotic lesions of endometriosis patients did not exhibit any differences in the Ki67 proliferation index. Our results demonstrate distinctive immunohistochemical variability between uterine remnants and endometriotic lesions in patients with MRKH syndrome compared with patients with endometriosis, indicating a possible explanation model of the yet-unknown etiology of endometriosis. For confirmation, investigation of a broader patient collective is necessary.

## 1. Introduction

Congenital MRKH syndrome is a Mullerian duct anomaly that is characterized by agenesis of the uterus and upper part of the vagina with normal secondary sex characteristics and normal female karyotype. It is the second most common cause of primary amenorrhea after gonadal dysgenesis and occurs in about 1 of 4500 female live births [1,2,3]. MRKH syndrome is classified as type 1, where an isolated uterovaginal aplasia can be diagnosed, or type 2 with associated extragenital malformations [4]. 

Uterine remnants can be seen in from 50% to almost 100% of all MRKH patients [5,6,7], whereas the endometrium can be histologically detected in up to 50% of all surgically removed remnants [5,8]. About one-half of these patients experience recurrent cyclic symptoms caused by proliferation of the endometrium, endometriosis, or myomas [8,9,10,11].

Endometriosis is defined as the existence of endometrial glands and stroma in extrauterine locations. It is an estrogen-dependent, inflammatory condition associated with pelvic pain and infertility [12].

To date a unifying theory of the origin of endometriosis is lacking. Generally theories can be classified as those promoting that endometriotic implants originate from uterine endometrium, whereas others state that implants originate from tissues other than the uterus [12]. MRKH syndrome with uterine remnants and functional endometrium is a rare subtype within the obstructive Mullerian duct anomalies. The etiology of MRKH syndrome is currently unknown. In a study by Brucker et al., endometrial stroma cells derived from patients with MRKH syndrome exhibited significantly less decidualization compared with healthy controls in addition to lower hormone responsiveness, possibly indicating a dysfunctional hormone receptor function that may play a role in the etiology of MRKH syndrome [13]. Furthermore, uterine remnants in patients with MRKH syndrome were found to contain typical uterine tissue with a more basalis-like endometrium and significantly lower proliferation compared with healthy controls [5].

Various studies have demonstrated the occurrence of endometriosis in patients with MRKH syndrome [14,15] and its clinical features [16].

To date, the histological features of endometriosis in patients with MRKH syndrome in comparison with healthy patients have not been elucidated.

We sought to analyze possible differences between the endometrium in uterine remnants and endometriotic lesions in patients with MRKH syndrome compared to samples from patients with endometriosis, to achieve new insights into the etiology and pathogenesis of MRKH syndrome and, possibly, endometriosis.

## 2. Material and Methods

Our study was a retrospective histological analysis of endometriotic tissue and endometrium of uterine remnants from patients with MRKH syndrome and their comparison to samples from patients with endometriosis. Tissue was obtained from patients with MRKH after informed consent. Patients underwent laparoscopically assisted creation of a neovagina using the modified Vecchietti technique (described elsewhere) at the Department of Obstetrics and Gynecology of the University of Tuebingen between 2010 and 2022 [17,18]. Patients with endometriosis simultaneously received a hysterectomy and removal of endometriotic tissue.

Correlation with the individual cycle phase was performed by obtaining standardized medical history and using hormone profiles from peripheral blood taken 1 day before surgery.

Every patient underwent a preoperative uro-MRI to exclude anomalies of the urogenital tract.

The study was approved by the Ethics Committee of Eberhard-Karls-University of Tuebingen (205/2014BO1).

### 2.1. Histologic Analysis

Endometriotic lesions and corresponding endometrial tissue were evaluated by an experienced gynecological pathologist. Macroscopic description was performed, and the sample was cut perpendicular to the longest axis. One section was cryopreserved, and the remaining specimen was fixed in formalin and embedded in paraffin. Sections of 2.5 μm were cut from each block and stained with hematoxylin and eosin (H & E).

### 2.2. Immunohistochemistry

Immunohistochemistry was performed using Ventana Discovery automated immunostaining system and Ventana reagents (Ventana Medical Systems). We took 2.5 μm-Sections from the respective specimen blocks and mounted them on Superfrost slides (Langenbrinck, Bern, Switzerland). Sections were deparaffinized with inorganic buffer. Pretreatment with ethylenediaminetetraacetic acid (EDTA) buffer was performed. EDTA-buffer was used pH 8.0 for estrogen receptor (ER), pH 6.0 for progesterone receptor (PR) and pH 8.4 for Ki67. Heat-induced epitope retrieval was performed for every antigen. Then, the primary antibody was applied for 1 h at room temperature (monoclonal rabbit anti-human ER, cone SP1, (DCS innovative Diagnostik Systeme, Hamburg, Germany); dilution 1:100, antibody diluent (Zytomed Systems, Berlin, Germany); monoclonal rabbit anti-human PR, clone SP2, (DCS innovative Diagnostik Systeme, Hamburg, Germany), dilution 1:150, antibody diluent DCS diluent; monoclonal mouse anti-human Ki-67-Antigen, clone MiB-1, M7240, (DakoCytomation, Glostrup, Denmark), dilution 1:400, antibody diluent Zytomed Systems, Berlin, Germany). A biotinylated detection kit including diaminobenzidine and horseradish peroxidase (DABMap Kit Ventana, Roche, Rotkreuz, Switzerland) was utilized. Counterstaining with hematoxylin and Blueing Reagent (Roche 760-2021) was performed. Slides were then washed and dehydrated with a series of ascending alcohol concentrations (40%, 70%, 96%) and covered with Cytoseal (Thermo Scientific, Waltham, MA, USA).

Nonexpressing tissues and staining protocols without the primary antibody were used as internal negative control.

### 2.3. Interpretation of Immunostaining Results

#### 2.3.1. Estrogen and Progesterone Receptor

ER and PR were quantified with the scoring system according to Remmele and Stegner [19]. The intensity of the nuclear stain was scored from 0 to 3 and then multiplied with a score for the number of positive cells (score 0 = 0, score 1 = 1–10%, score 2 = 11–50%, score 3 = 51–80%, score 4 = 81–100%), producing a score from 0 to 12.

#### 2.3.2. MiB1 (Antibody against Ki67)

The percentage of positive nuclei was estimated by evaluation of at least 100 cells in one distinct area in the respective compartment of the specimen.

### 2.4. Hormone Profile and Correlation with Cycle Phase

Whole blood collection from patients with MRKH and endometriosis was performed 1 day before or after surgery. Estrogen, progesterone, luteinizing hormone (LH) and follicle-stimulating hormone (FSH) was measured in blood serum using a chemiluminescence immunoassay (Vitros eci, Madrid, Spain; Diagnostic Product Coorperation).

The proliferative phase (cycle phase 1) was assigned when progesterone was <2.5 ng/mL, and the secretory phase (cycle phase 2) when progesterone was >5 ng/mL and the LH/FSH ratio was >1.5.

## 3. Results

Of the 319 patients who underwent neovagina creation between 2010 and 2021 and the additional removal of uterine remnants, endometriotic tissue was detected in 9 patients (3.1%).

Median age of patients at time of surgery was 23.5 years (IQR 18.25–32).

Adenomyosis was diagnosed within the uterine remnants in five patients; adenomyosis and endometriotic peritoneal lesions were found in one patient; one patient was diagnosed with endometriosis in the peritoneum, ovary and remnants; endometriosis was detected in the ovaries in two patients.

None of the patients had associated malformations. Three patients reported cyclic abdominal pain.

Uterine remnants were located bilaterally in all patients. One patient had previously undergone removal of remnants. Hematometra due to endometrial proliferation was not diagnosed in any of the patients. In one patient with a severe endometriosis and prominent remnants, the fallopian tubes were altered similarly to a hemato- or sactosalpinx.

The cycle phase was evaluated by obtaining a hormonal profile from peripheral blood taken 1 day before or after surgery. Six patients were in cycle phase 1 (proliferative phase), one patient was in cycle phase 2 (secretory phase), and the cycle phase was unknown in three patients (for details see Table 1).

The expression of hormonal receptors was evaluated in the uterine remnants of five patients, as endometrial specimens from the remaining patients were too small for immunohistochemistry. All specimens exhibited a high expression of estrogen and progesterone receptor (IRS 12). In the corresponding endometriotic lesions, expression of hormonal receptors was more heterogeneous. The expression of progesterone receptor was lower in epithelial cells than in stromal cells in the two ovarian endometriotic specimens. In two patients, estrogen-receptor expression in the endometriotic tissue was comparably lower than in the corresponding uterine remnants.

The proliferation marker Ki67 differed significantly in two patients when comparing the proliferation in the endometriotic tissue to the remnants, as Mib was significantly lower in the remnants than in the endometriotic specimens (*p* = 0.0254). Both patients were in the proliferative phase. In two patients, Ki67 was <1% in both the endometriotic tissue and uterine remnants (for details, see Table 2).

**Figure 1:**
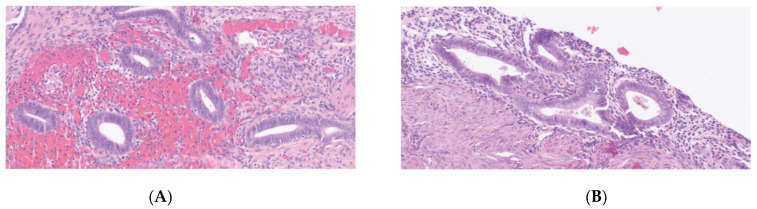
Hematoxylin and eosin (H&E) staining (×200). (**A**) endometriosis. (**B**) uterus remnant.

**Figure 2:**
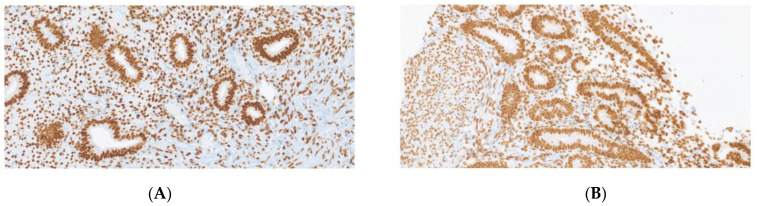
Estrogen receptor (ER) with strong nuclear expression (×200). (**A**) endometriosis. (**B**) uterus remnant.

**Figure 3:**
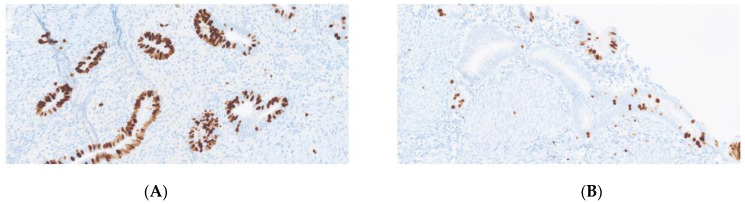
Immunohistochemistry for K67 (MiB1), demonstrating higher proliferative index in endometriosis than in the uterine remnant (×200). (**A**) endometriosis. (**B**) uterus remnant.

**Figure 4:**
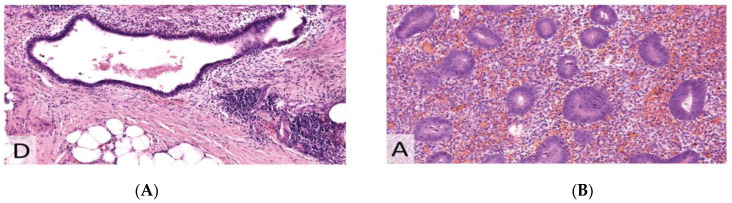
Hematoxylin and eosin (H&E) staining (×200). (**A**) Endometriosis. (**B**) Endometrium.

**Figure 5:**
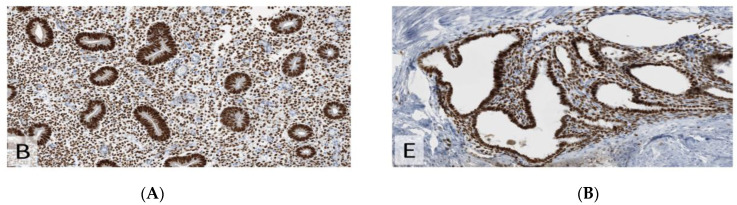
Estrogen receptor (ER) with strong nuclear expression (IRS 12) (×200). (**A**) Endometriosis. (**B**) Endometrium.

**Figure 6:**
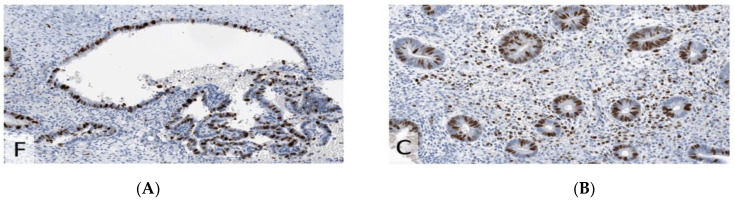
IHC for Ki67 (MiB1), demonstrating the same proliferative index (40%) in endometriosis compared to endometrium (×200). (**A**) Endometriosis. (**B**) Endometrium.

**Figure 7:**
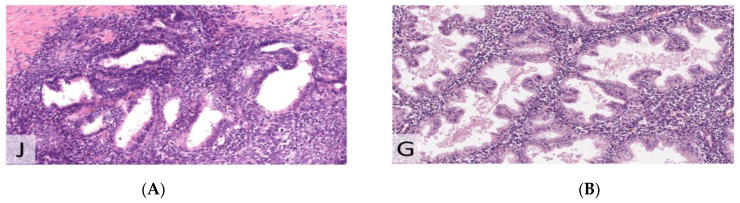
Hematoxylin and eosin (H&E) staining (×200). (**A**) Endometriosis. (**B**) Endometrium.

Endometriotic tissue and the corresponding endometrium were analyzed in two patients with endometriosis. The two patients were 37 and 39 years old and both were premenopausal. They did not take any hormonal therapy. One patient presented with endometriosis of the bladder and subsequent partial removal of the bladder during surgery. The other patient had extensive peritoneal endometriosis (rASRM stage 4). One patient was in the proliferative and one in the secretory cycle phase at the timepoint of surgery. Both the endometrial and the endometriotic tissue of the patient in the proliferative phase exhibited increased expression of the estrogen-receptor (IRS 12). Ki67 was 40% both in the endometrial and in endometriotic tissue. Samples from the patient in the secretory phase demonstrated a heterogenous moderate-to-strong expression of the estrogen-receptor in 75% of the epithelium, in both endometrial and endometriotic tissue. Ki67 was 0% in both the endometriotic and endometrial tissue (for details, see Figure 1, Figure 2, Figure 3, Figure 4, Figure 5, Figure 6 and Figure 7).

Comparison of uterus remnant and endometriotic specimen in a patient with MRKH (Figure 1, Figure 2 and Figure 3). 

Comparison of uterine endometrium and endometriotic specimen in healthy patients. Patient in proliferative phase (Figure 4, Figure 5 and Figure 6).

**Figure 8:**
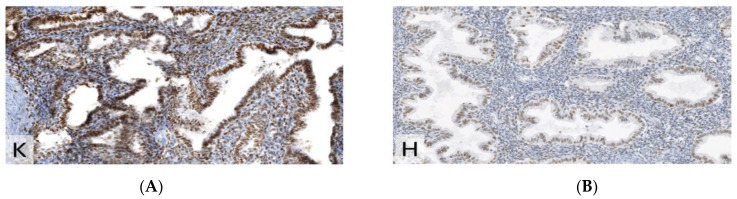
Estrogen receptor (ER) with heterogeneous moderate to strong expression in 75% of the epithelium (×200). (**A**) Endometriosis. (**B**) Endometrium.

**Figure 9:**
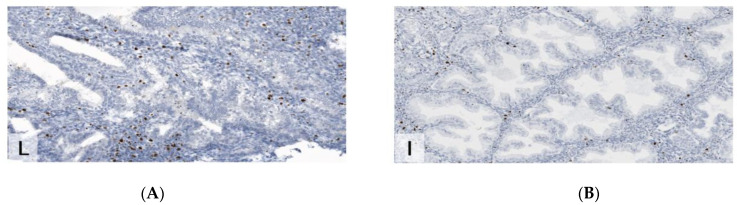
IHC for Ki67 (MiB1), demonstrating the same proliferative index (lower than in proliferative phase) in endometriosis compared to endometrium (×200). (**A**) Endometriosis. (**B**) Endometrium.

Patient in secretory phase (Figure 7, Figure 8 and Figure 9).

## 4. Discussion

To date, no study has investigated and compared the immunohistochemistry of uterine remnants and corresponding endometriotic lesions in MRKH patients with endometriotic samples of healthy patients. In our findings, there was no significant quantitative difference in the expression of hormonal receptors in endometriotic tissue and in the endometrium of uterine remnants in MRKH patients. Therefore, it remains unclear why the endometrium of the remnants was mostly dormant. This observation may possibly underline the hypothesis of deficient hormonal receptors in patients with MRKH syndrome [20].

Interestingly, in two patients with MRKH syndrome in our study, the Ki67 proliferation index was significantly higher in the peritoneal endometriotic lesion than in the uterine rudiments. This might suggest a differing origin of the endometriotic tissue compared to the uterine endometrium.

This is further underlined by the fact that, in contrast with the samples from MRKH patients, endometrial and endometriotic tissue from endometriosis patients did not exhibit any differences regarding immunohistochemical evaluation of the proliferation index and were concordant and adequate for the respective cycle phase.

Throughout history, obstructive Mullerian duct anomalies have served as an explanatory model to support the theory of retrograde menstruation, as an increased incidence of endometriosis was noted in patients with associated hematocolpos or hematometra [21].

Data on endometriosis in MRKH patients are mainly based on case reports; therefore, demographic data and incidence rates are sparse [8,14,22,23,24,25,26,27,28,29]. Considering the previous considerations, we assumed that the incidence of endometriosis in patients with MRKH syndrome and functioning endometrium would be at least as high as it was in the general population. Here, endometriosis is estimated to affect 10–15% of all women of reproductive age [30]. In contrast to general findings, the incidence of endometriosis in our patient collective was considerably lower.

In the aforementioned case reports, the authors reported mostly laparoscopically detected endometriomas, peritoneal lesions or adenomyosis; in some cases, the absence of functional endometrium in uterine remnants or a complete lack of uterine rudiments was reported.

These findings may emphasize the theory of coelomic metaplasia as a complementary factor in the development of endometriosis [31].

In contrast, Konrad et al. emphasized, in their review of endometriosis in MRKH patients, that many of these published case reports are missing evidence of the existence of uterine remnants, as diagnostics for uterine remnants are inconsistently based on MRI-diagnostics or two-dimensional ultrasound and rarely based on histological evidence [32]. Histological proof of endometriotic lesions was presented in only a few articles [22,25,26,31,33]. Konrad et al. highlighted that the existence of functional endometrial tissue in uterine remnants is essential for the development of endometriosis in MRKH patients. As endometriomas were the most common manifestation in these patients, the authors stated that coelomic metaplasia may have contributed to their development [32].

Therefore, the surgical removal of uterine remnants with functional endometrial tissue is recommended to minimize the risk of developing endometriosis or reduce its extent linked to retrograde menstruation [34].

In our study, six patients had histological proof of the existence of dormant endometrium in their rudiments, one patient presented with adenomyosis-like endometrial glands, and one patient had extensive prismatic epithelium within the remnant. Only one patient from our cohort, who was diagnosed with severe endometriosis, had proliferative endometrium in a uterine remnant. As all patients underwent blood collection for hormonal profiling at the time of surgery, it is surprising that almost all patients were in the first cycle phase; only one patient with dormant endometrium had a hormonal profile consistent with the second cycle phase.

The existence of endometrioma was reported in more than half of cases in the literature. Only two out of nine patients were diagnosed with endometrioma in our study. Diagnosis was made prior to surgery, as all patients received a preoperative MRI of the urogenital tract.

One limitation of our study is the small sample size. Due to the rarity of MRKH syndrome, it is challenging to establish a cohort with an adequate number of patients, even though our department can refer to the largest collective of patients with MRKH syndrome in Germany. Therefore, a future approach might be the implementation of international multi-center studies with standardized diagnostic criteria and histological evaluation to provide a larger patient cohort. Moreover, the comparison of endometriosis to more patients with endometriosis without Mullerian anomaly might produce relevant differences and potentially highlight new explanation models for the development of endometriosis, possibly promoting an extrauterine origin of the endometriotic implants.

## 5. Conclusions

Our results revealed significant immunohistochemical variability between uterine remnants and endometriotic lesions in patients with MRKH syndrome compared with samples from patients with endometriosis without Mullerian anomaly, indicating a possible explanation model for the yet-unknown etiology of endometriosis. Despite the increased expression of hormonal receptors in the endometrial tissue of remnants, the endometrium mainly remained dormant, even in the proliferative cycle phase, possibly underlining the hypothesis of deficient hormonal receptors in patients with MRKH syndrome. To confirm our findings, an international multi-center study with a standardized diagnostic procedure and sample collection is needed to estimate the true incidence of endometriosis in MRKH syndrome.

## Figures and Tables

**Table 1:** Clinical characteristics.

Patient Number	Age at Surgery	Existing Uterus Remnants	Adenomyosis	Localization of Endometriosis	Clinical Symptoms	Associated Malformations	Hormonal Profile
1	16	Both sides	No	Right ovary	Abdominal pain	None	N/A
1	23	Removed	/	Right ovary	Unknown	None	N/A
2	49	Both sides	Yes	Adenomyosis	None	None	N/A
3	16	Both sides	No	Peritoneum	None	None	Cycle phase 1
4	19	Both sides	Yes (left side)	Adenomyosis	None	None	Cycle phase 2/periovulatory
5	24	Both sides	Yes	Peritoneum	Cyclic abdominal pain	None	Cycle phase 1
6	27	Both sides	Yes (left side)	Adenomyosis	None	None	Cycle phase 1
7	29	Both sides	Yes (right side)	Adenomyosis	Unknown	None	Cycle phase 1
8	20	Both sides	Yes (left side)	Adenomyosis	Cyclic abdominal pain	None	Cycle phase 1
9	41	Both sides	Yes (both sides)	Peritoneum	None	None	Cycle phase 1

N/A, not available.

**Table 2:** Results for immunohistochemistry IRS (ER, PR) and Ki67.

Patient Number	Estrogen Receptor Expression Uterus Remnants	Progesterone Receptor Expression Uterus Remnants	Estrogen Receptor Expression Endometriotic Lesion	Progesterone Receptor Expression Endometriotic Lesion	Ki67 Uterus Remnants	Ki67 Endometriotic Lesion
1	12	12	6	4 (stroma 12)	<1%	<1%
1	N/A	N/A	9	4 (stroma 12)	N/A	8%
2	12	9	N/A	N/A	N/A	N/A
3	12	12	4	12	15%	75%
4	12	12	N/A	N/A	5%	N/A
5	N/A	N/A	N/A	N/A	N/A	N/A
6	N/A	N/A	N/A	N/A	N/A	N/A
7	N/A	N/A	N/A	N/A	N/A	N/A
8	12	12	12	9	<1%	1%
9	12	12	12	12	15%	80%

N/A, not available.

## Data Availability

The data presented in this study are available on request from the corresponding author. The data are not publicly available due to patient confidentiality.
